# A Bayesian Generative Model for Surface Template Estimation

**DOI:** 10.1155/2010/974957

**Published:** 2010-09-20

**Authors:** Jun Ma, Michael I. Miller, Laurent Younes

**Affiliations:** ^1^Center for Imaging Science, Department of Biomedical Engineering, The Johns Hopkins University, 320 Clark Hall, Baltimore, MD 21218, USA; ^2^Center for Imaging Science, Department of Biomedical Engineering, The Johns Hopkins University, 301 Clark Hall, Baltimore, MD 21218, USA; ^3^Center for Imaging Science, Department of Applied Math and Statistics, The Johns Hopkins University, 3245 Clark Hall, Baltimore, MD 21218, USA

## Abstract

3D surfaces are important geometric models for many objects of interest in image analysis and Computational Anatomy. In this paper, we describe a Bayesian inference scheme for estimating a template surface from a set of observed surface data. In order to achieve this, we use the geodesic shooting approach to construct a statistical model for the generation and the observations of random surfaces. We develop a mode approximation EM algorithm to infer the maximum a posteriori estimation of initial momentum *μ*, which determines the template surface. Experimental results of caudate, thalamus, and hippocampus data are presented.

## 1. Introduction

3D surfaces are important geometric models for many objects of interest in image analysis and Computational Anatomy. For example, they are often used to represent the shape of 3D objects, the surface of human faces, and the boundaries of brain structures or of other human organs. Most data analysis methods in this domain are template-centered, and a proper estimation of a template plays an important role to obtain high quality results. This paper is devoted to the description of statistically supported template estimation method which is adapted to surface data sets.

Our approach will be to build a generative statistical shape model in which the template is a parameter. We will then estimate it using maximum likelihood. This model relies on the very natural setting for which an observed surface is a noisy version of a random deformation of the template. This is the most generic and most basic approach of the deformable template paradigm, even if we add a small refinement by including a prior distribution on the template, based on what we will call a *hypertemplate*. Even with this global scheme which is fairly simple, we will see that implementing it in the context of surfaces will constitute a significant theoretical and numerical challenge.

At the exception of the recent work of [1], this approach significantly differs from what has been mostly proposed in the literature, in which most of the methods compute templates as averages over specific common parametrizations of the surfaces (using, for example, the sphere as a parameter space [2]). Parametric representations, however, are limited by the fact that, because they are defined a priori and independently for each object, they cannot be assumed to suitably align important features in a given data set of surfaces (i.e., give similar coordinates to similar features in the surfaces). This usually results in oversmoothed template surfaces (which is the equivalent of getting blurry template images in the case of image averaging). In [1], a similar diffeomorphic transformation model is used, but, as we will see, our Bayesian construction will provide a well-specified template whereas [1] needs to rely to topologically unconstrained approximations to end up with a manageable template.

In addition to the references above, there have been several publications addressing the issue of shape averaging over a dataset, although most of them work with 3D volume data or landmark points set. In several cases, the average is based on metric properties of the space of shapes [3–7], and the template is computed as an intrinsic average, minimizing the sum of square distances to each element of the set (Fréchet mean). Such methods have been implemented in the context of diffeomorphic deformation models (which are also models of interest in the present paper) for landmark matching [8], for 3D average digital atlas construction [9], and to quantify variability in heart geometry [10]. Other definitions of the average, adapted to situations in which the data is corrupted by noise, have been proposed [11–13], based on variational approaches (but not relying on a generative statistical model).

Our approach to build a generative statistical shape model is reminiscent of the construction developed in [14] for linear models of deformations, and in [15] for large diffeomorphic deformations in 3D volume image averaging. Adapting these ideas to surfaces will however require new algorithms and numerical developments.

In order to present our model, we need to first provide some background materials and notation, describing in particular the geodesic shooting equations that we will use to generate deformed surfaces. We will then introduce a random statistical model describing the generation and the observations of random surfaces. We then develop a Mode Approximation EM algorithm for surface averaging, to estimate the template from observations. In the optimization part, we derive and implement a new variational scheme, which is also applicable to surface matching, providing an alternative approach to the one originally proposed in [16, 17]. Finally, we present and discuss experimental results on caudate, thalamus, and hippocampus data.

## 2. EPDiff for Surface Evolution

We will base our random shape model on the so-called EPDiff equation, which describes the evolution of deformable structures (like images, surfaces, or landmarks) under the action of groups of diffeomorphisms. It is a geodesic equation for a Riemannian metric on diffeomorphisms, and describes a momentum conservation law in the associated Hamiltonian system. The reader interested by the theory behind this equation can refer to [18–20], but most of this background will not be needed for the present paper, in which we will only use the specific form of the equations for surface evolution. The term EPDiff comes from its determination as an Euler-Poincaré equation in the group of diffeomorphisms, as introduced in [21]. One of its main interests here is that it provides a numerically stable, easily described, Hamiltonian evolution over diffeomorphisms, which will constitute an ideal support for our shape models.

The EPDiff equations describe the combined time evolution of a diffeomorphism, denoted *ϕ*(*t*, ·) and of what can be interpreted as a momentum, denoted *p*(*t*, ·). The initial conditions are always *ϕ*(0, *x*) = *x* for *ϕ*, and some initial value, *p*_0_, for *p*. This *initial momentum* will be a key component of the statistical model that will be built later on.

Let us start with the simplest form of the equation, which assumes that *p*_0_ is a vector-valued function over ℝ^*d*^, that is, *p*_0_ : ℝ^*d*^ → ℝ^*d*^. It involves a smoothing kernel, *K*, defined on ℝ^3^ × ℝ^3^, a typical choice being

(1) $$\begin{array}{c} K\left(x,y\right)=\text{cst}\;\exp\;\left(-\frac{{||x-y||}^{2}}{2\tau^{2}}\right). \end{array}$$

Letting ∇_1_ *K* denote the gradient of *K* with respect to its first variable, the corresponding EPDiff equation is

(2) $$\begin{array}{c} \frac{d\phi }{dt}\left(t,x\right)=\int_{{\mathbb{R}}^{3}}K\left(\phi \left(t,x\right),\phi \left(t,y\right)\right)p\left(t,y\right)dy,\\ \frac{dp}{dt}\left(t,x\right)=-\int_{{\mathbb{R}}^{3}}\nabla_{1}K\left(\phi \left(t,x\right),\phi \left(t,y\right)\right)\left(p\left(t,x\right)\cdot p\left(t,y\right)\right)dy. \end{array}$$

Here, the notation *a* · *b* refers to the usual dot product between vectors in ℝ^3^. If *K* is smooth enough, this system has solutions for arbitrary large *t*, and the mapping *x* ↦ *ϕ*(*t*, *x*) is a diffeomorphism at all times.

The interesting fact about these equations is that they can have singular variants that are described in a similar way and have the same existence properties. The simplest way to relate the variants to the previous equation is to replace the Lebesgue's measure in the integrals by another, possibly singular, measure. For example, taking a surface *S*_0_ in ℝ^3^, we can use the volume form on *S*_0_ as a reference measure and obtain the equations (in which *p*_0_ and *p*(*t*, ·) need only to be defined on *S*_0_)

(3) $$\begin{array}{cc} \frac{d\phi }{dt}\left(t,x\right) & =\int_{S_{0}}K\left(\phi \left(t,x\right),\phi \left(t,y\right)\right)p\left(t,y\right)d\omega_{S_{0}}\left(y\right),\\ \frac{dp}{dt}\left(t,x\right) & =-\int_{S_{0}}\nabla_{1}K\left(\phi \left(t,x\right),\phi \left(t,y\right)\right)\\ & \;\;\;\;\times \left(p\left(t,y\right)\cdot p\left(t,y\right)\right)d\omega_{S_{0}}\left(y\right), \end{array}$$

where *d* *ω*_*S*_0__ is the volume form on *S*_0_. Note that the first equation is defined for *x* ∈ ℝ^3^, but it suffices to use it for *x* ∈ *S*_0_ to obtain an equation for the evolving surface

(4) $$\begin{array}{c} S_{t}=\phi \left(t,S_{0}\right)=\left\{y=\phi \left(t,x\right),x\in S_{0}\right\}. \end{array}$$

We can write a discrete form of the equations by replacing *d* *y* by a sum of Dirac measures (at points *x*_1_,…, *x*_*L*_ in ℝ^3^), which gives, letting *a*_*l*_(*t*) = *p*(*t*, *x*_*l*_),

(5) $$\begin{array}{c} \frac{d\phi }{dt}\left(t,x\right)=\overset{L}{\underset{l=1}{\sum }}K\left(\phi \left(t,x\right),\phi \left(t,x_{l}\right)\right)a_{l},\\ \frac{da_{k}}{dt}=-\overset{L}{\underset{l=1}{\sum }}\left(a_{l}\cdot a_{k}\right)\nabla_{1}K\left(\phi \left(t,x_{k}\right),\phi \left(t,x_{l}\right)\right). \end{array}$$

Similarly to (3), the first equation is valid for all *x* ∈ ℝ^3^, but it suffices to solve it for *x* = *x*_*l*_, *l* = 1,…, *L* to obtain the evolution of the point set

(6) $$\begin{array}{c} x_{l}\left(t\right)=\phi \left(t,x_{l}\right). \end{array}$$

Also, (5) can be seen as a discretization of (3) in which *x*_1_,…, *x*_*L*_ are the vertices of a triangulation of *S*_0_, and *a*_*l*_(*t*) = *p*(*t*, *x*_*l*_)*δ* *σ*_*S*_0__(*x*_*l*_), where *δ* *σ*_*S*_0__(*x*_*l*_) is the area of a surface element around *x*_*l*_.

The evolution of point sets is the most important from a practical point of view, since it is an ODE that can be easily implemented. Assuming a radial kernel *K*(*x*, *y*) = *γ*(||*x*−*y*||^2^) like in (1), and denoting *γ*_*kl*_ = *γ*(||*x*_*k*_−*x*_*l*_||^2^), and *γ*_*kl*_′ = *γ*′(||*x*_*k*_−*x*_*l*_||^2^), (5) can be rewritten as

(7) $$\begin{array}{c} \frac{\text{d}x_{k}}{\text{d}t}=\overset{L}{\underset{l=1}{\sum }}\gamma_{kl}a_{l},\;\;\frac{\text{d}a_{k}}{\text{d}t}=-2\overset{L}{\underset{l=1}{\sum }}\gamma_{kl}^{\prime }\left(a_{l}\cdot a_{k}\right)\left(x_{k}-x_{l}\right). \end{array}$$

Once the initial position of the vertices, *x*(0) = (*x*_1_(0),…, *x*_*L*_(0)), and the initial momentum, *a*(0) = (*a*_1_(0),…, *a*_*L*_(0)), are provided, the evolution of the point set is uniquely determined.

## 3. Generative Model for Surface Observation

### 3.1. Random Triangulated Surfaces

If a triangulated template surface *T* with vertices *x* ^(*T*)^ is given, and we solve, until time *t* = 1, (5) initialized with *x*(0) = *x* ^(*T*)^ and a random initial momentum *a*(0) = *α*, the displaced vertices provide a random perturbation of the initial surface that will be denoted by *T*_*α*_. This is stated in the following definition.

**Definition 1**

Let *T* be a triangulated surface with vertices *x* ^(*T*)^ = (*x*_1_^(*T*)^,…, *x*_*L*_^(*T*)^). Let *α* ∈ (ℝ^3^)^*L*^ be a collection of *L* vectors in ℝ^3^. Let (*x*(*t*), *a*(*t*)) be the solution of (5) with initial condition *x*(0) = *x* ^(*T*)^ and *a*(0) = *α*. One defines *T*_*α*_ to be the triangulated surface with vertices *x* ^(*T*_*α*_)^ = *x*(1) and the same topology as *T*.

By letting *α* be random, we build *T*_*α*_ as a random deformation of *T*. This will form the “ideal”, unobserved, surface, of which only a noisy version is observable (the noise process will be described in the next section).

Following [15], we will use a Bayesian formulation in which *T* is itself represented as a random deformation *T*_0,*μ*_ : = (*T*_0_)_*μ*_, where *T*_0_ is a fixed surface that we will call the hypertemplate, and *μ* is a prior initial momentum shooting from *T*_0_ to *T* (same notation as in Definition 1). One of the main interests of using a hypertemplate is to fix the topology of *T* so that it belongs, by construction, to the same class of objects as *T*_0_.

So, if *N* surfaces are observed, we need to model the probability distribution of the prior momentum, *μ* (starting at *T*_0_), which specifies *T* = *T*_0,*μ*_ and of *N* deformation momenta *α* ^(1)^,…, *α* ^(*N*)^ which specify the surfaces *T*_*α*^(1)^_,…, *T*_*α*^(*N*)^_. We now provide a statistical model for the joint probability distribution of *μ*, *α* ^(1)^,…, *α* ^(*N*)^.

We first introduce some notation. Letting *K* be the kernel introduced in the previous section to define the geodesic shooting equations, we let Γ_*T*_ be the 3*L* by 3*L* matrix formed with the 3 by 3 blocks *K*(*x*_*k*_^(*T*)^, *x*_*l*_^(*T*)^)*I* *d*_ℝ^3^_. We define, for a triangulated surface *T* with *L* vertices *x* ^(*T*)^, and *α* ∈ ℝ^3*L*^,

(8) $$\begin{array}{c} {||\alpha ||}_{T}^{2}=\alpha^{*}\Gamma_{T}\alpha =\overset{L}{\underset{k,l=1}{\sum }}K\left(x_{k}^{\left(T\right)},x_{l}^{\left(T\right)}\right)\left(\alpha_{k}\cdot \alpha_{l}\right). \end{array}$$

We define the joint distribution of *μ*, *α* ^(1)^,…, *α* ^(*N*)^ on ℝ^3*L*^ × (ℝ^3*L*^)^*N*^ by

(9) $$\begin{array}{c} p\left(\mu ,\alpha^{\left(1\right)},\ldots ,\alpha^{\left(N\right)}\right)=\frac{1}{Z}\exp\;\left(-\frac{1}{2}\lambda {||\mu ||}_{T_{0}}^{2}-\frac{1}{2}\overset{N}{\underset{n=1}{\sum }}{||\alpha^{\left(n\right)}||}_{T_{0,\mu }}^{2}\right), \end{array}$$

where *λ* is a fixed parameter regulating the weight on the hypertemplate.

There is a technical difficulty here, which is that one must make sure that this probability can be normalized (*Z* exists), which requires that the exponential is integrable. That this is true is not straightforward, and we have not been able to find a proof that works with any choice of the kernel *K*. One way to deal with this is to introduce a constant *A*_*μ*_ (which can be chosen arbitrarily large so that it does not interfere with the algorithms that will follow), and add to the model the constraint that ||*μ*||_*T*_0__ is smaller than *A*_*μ*_. Under such an assumption, one obtains (after integrating out the *α*'s)

(10) $$\begin{array}{c} Z={\left(2\pi \right)}^{3NL/2}\int_{{||\mu ||}_{T_{0}}\leq A_{\mu }}\exp\;\left(-\frac{1}{2}{||\mu ||}_{T_{0}}^{2}-\frac{N}{2}\log\;\;\det\;\;\Gamma_{T_{0,\mu }}\right)d\mu . \end{array}$$

This is finite, since, for any given *μ*, the transformation *x* ^(*T*_0_)^ → *x* ^(*T*_0,*μ*_)^ is the restriction of a diffeomorphism to the vertices of *T*_0_ (as seen from (5)). This implies that Γ_*T*_0,*μ*__ is nonsingular, and its determinant is bounded away from 0 when *μ* is restricted to a compact space.

In fact, the choice *A*_*μ*_ = *∞* can be proved to be acceptable for a large class of kernels. Those are kernels for which the smallest eigenvalue of Γ_*T*_ decreases at a speed which is at most polynomial in the minimal distance, *h*_*T*_, between the vertices. A list of kernels satisfying this property can be found in [22]. For such kernels, we find that (*L* being fixed) log det Γ_*T*_ = *O*(log *h*_*T*_). Just sketching the argument here, one can prove, using elementary properties of dynamical systems, that *h*_*T*_ = *O*(exp (−*C*||*μ*||_*T*_0,*μ*__) for some constant *C*, so that the log determinant in (9) is linear in ||*μ*||_*T*_0,*μ*__ and *Z* is well defined, even with *A*_*T*_0,*μ*__ = *∞*. For very smooth kernels, including the Gaussian, bounds on the smallest eigenvalue of Γ_*T*_ are much worse (with a decay which is exponential in (−1/*h*_*T*_^2^)), and the previous argument does not work. Since the bounds in [22] hold uniformly with respect to the number of points, a polynomial bound may still be valid for a fixed *L*, although we were unable to discover it.

**Figure 1:**
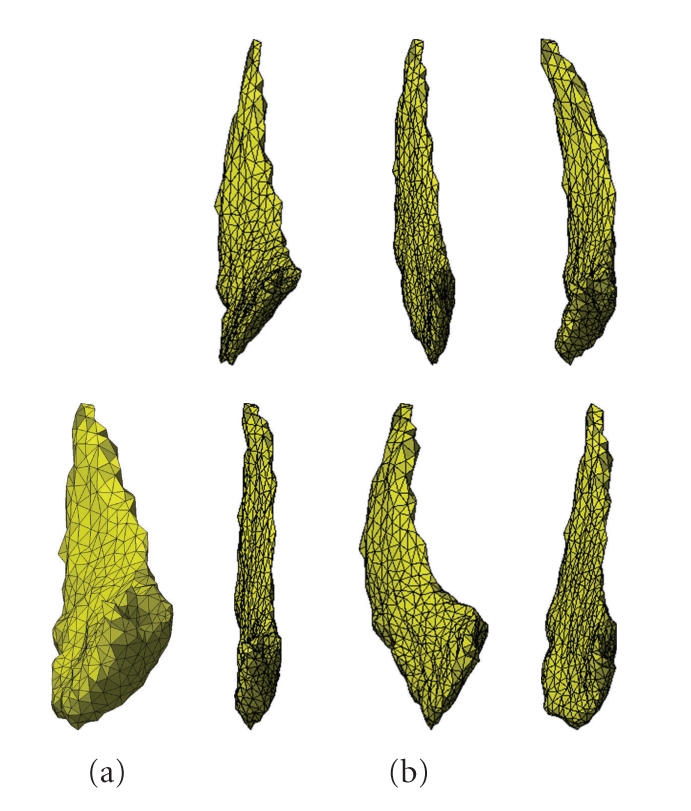
Random deformation of a template caudate surface (left). The six surfaces following the template are independent realization of the model described in (9).

Notice that, conditionally to the template, the momenta *α* are independent and follow a Gaussian distribution with inverse covariance matrix given by Γ_*T*_. An example of simulated random deformations obtained using such a model is provided in Figure 1.

### 3.2. Observation and Noise

The second part of our generative model is to describe the observation process, which takes an ideal surface *T*_*α*_ generated according to the model above, and returns the noisy observable.

Modeling such a noise process is a tricky issue. Obvious choices (like adding noise to the vertices of *T*_*α*_) do not work because one cannot assume that the observed surfaces are discretized consistently with the template. In this paper, we will work around this issue by assimilating the observation of a surface that of a singular Gaussian process.

For this, we consider that surfaces in ℝ^3^ are not observable directly, but only via their action on test functions, that we will call *sensors*. We define a sensor to be a smooth vector field *w* over ℝ^3^ (typically with small support). Given an oriented surface *S*, define

(11) $$\begin{array}{c} \left(S,w\right)=\int_{S}w\left(s\right)\cdot N_{S}\left(s\right)d\omega_{S}\left(s\right), \end{array}$$

where *N*_*S*_ is the normal to *S*. The real number, (*S*, *w*) is the measurement of *S* through the sensor *w*.

Now, modeling noisy surfaces will result in assuming that, given any *w*, the measurement (*S*, *w*) is a random variable. We will assume that it is Gaussian, and more generally, that, given *m* sensors, *w*_1_,…, *w*_*m*_, the random vector ((*S*, *w*_*j*_), *j* = 1,…, *m*) is Gaussian.

*S*, via its action on sensors, is therefore modeled as a Gaussian random field. Given an ideal surface *T*_*α*_, we will assume that its mean is given by

(12) $$\begin{array}{c} E\left(\left(S,w\right)\right)=\left(T_{\alpha },w\right) \end{array}$$

and the process is thereafter uniquely characterized by its covariance operator

(13) $$\begin{array}{c} G\left(w,\tilde{w}\right)=\mathrm{cov}\;\left(\left(S,w\right),\left(S,\tilde{w}\right)\right). \end{array}$$

We will assume that this covariance is associated to a symmetric operator *L*_obs_ so that

(14) $$\begin{array}{c} G\left(w,\tilde{w}\right)=\sigma^{2}\int_{{\mathbb{R}}^{d}}\left(L_{\text{obs}}w\left(x\right)\cdot \tilde{w}\left(\tilde{x}\right)\right)dx\;d\tilde{x}. \end{array}$$

(The apparently redundant parameter *σ* ^2^, which could have been included in *L*_obs_, appears here because it can be easily estimated by the algorithm, with the operator *L*_obs_ remaining constant.)

To finalize our model, it remains to describe how *S* is discretized, that is, to make explicit a finite family of sensors through which *S* is measured. Let (*z*_*j*_, *j* ∈ *J*) form a regular grid of points in *Ω*. Let *γ*_*s*_ be a radial basis function (a Gaussian, e.g.,) and define, for *j* ∈ *J* and *d* = 1,2, 3

(15) $$\begin{array}{c} w_{j,d}=\gamma_{s}\left(x-z_{j}\right)e_{d} \end{array}$$

where *e*_*d*_ is the *d*th vector of the standard basis of ℝ^3^ (this therefore specifies 3|*J*| sensors). The resulting observed variables are

(16) $$\begin{array}{c} y_{j,d}=\left(S,w_{j,d}\right)=\int_{S}\gamma_{s}\left(s-z_{j}\right)N_{S}^{\left(d\right)}\left(s\right)d\omega_{S}\left(s\right), \end{array}$$

where *N*_*S*_^(*d*)^ = *N*_*S*_ · *e*_*d*_ is the *d*th coordinate of *N*_*S*_. These variables are, by assumption, jointly Gaussian, with means *m*_*j*,*d*_ = (*T*_*α*_, *w*_*j*,*d*_) and covariance matrix

(17) $$\begin{array}{cc} g_{i,d,i^{\prime },d^{\prime }} & =G\left(w_{i,d},w_{i^{\prime },d^{\prime }}\right)\\ & =\sigma^{2}\delta_{d,d^{\prime }}\int_{{\mathbb{R}}^{d}}L_{\text{obs}}\gamma_{s}\left(x-z_{i}\right)\gamma_{s}\left(\tilde{x}-z_{i^{\prime }}\right)dx\;d\tilde{x}. \end{array}$$

Assuming that *L*_obs_ is translation invariant, the resulting expression is a function of *z*_*i*_ − *z*_*i*′_ that we will denote

(18) $$\begin{array}{c} g_{i,d,i^{\prime },d^{\prime }}=\sigma^{2}\delta_{dd^{\prime }}\gamma_{\text{obs}}\left(z_{i}-z_{i^{\prime }}\right). \end{array}$$

Let *R*_obs_ = (*r*_*ij*_^(obs)^) be the inverse matrix of the one with coefficients (*γ*_obs_(*z*_*j*_ − *z*_*j*_′), *j*, *j*′ ∈ *J*). The log likelihood of the process will include error terms taking the form

(19) $$\begin{array}{c} {ℰ}_{\text{obs}}=\frac{1}{\sigma^{2}}\underset{j,j^{\prime }\in J}{\sum }\;\overset{3}{\underset{d=1}{\sum }}r_{j,j^{\prime }}^{\left(\text{obs}\right)}\left(y_{j,d}-\left(T_{\alpha },w_{j,d}\right)\right)\left(y_{j^{\prime },d}-\left(T_{\alpha },w_{j^{\prime },d}\right)\right). \end{array}$$

Replacing *y*_*j*,*d*_ by its expression in (16), we have

(20) $$\begin{array}{cc} \sigma^{2}{ℰ}_{\text{obs}} & =\underset{j,j^{\prime }\in J}{\sum }\;‍\overset{3}{\underset{d=1}{\sum }}r_{j,j^{\prime }}^{\left(\text{obs}\right)}\int_{S}\gamma_{s}\left(x-z_{j}\right)N_{S}^{\left(d\right)}\left(x\right)d\omega_{S}\left(x\right)\\ & \;\times \int_{S}\gamma_{s}\left(x-z_{j^{\prime }}\right)N_{S}^{\left(d\right)}\left(x\right)d\omega_{S}\left(x\right)\\ & \;-2\underset{j,j^{\prime }\in J}{\sum }r_{j,j^{\prime }}^{\left(\text{obs}\right)}\overset{3}{\underset{d=1}{\sum }}\int_{S}\gamma_{s}\left(x-z_{j}\right)N_{S}^{\left(d\right)}\left(x\right)d\omega_{S}\left(x\right)\\ & \;\times \int_{T_{\alpha }}\gamma_{s}\left(x-z_{j^{\prime }}\right)N_{T_{\alpha }}^{\left(d\right)}\left(x\right)d\omega_{T_{\alpha }}\left(x\right)\\ & \;\times \underset{j,j^{\prime }\in J}{\sum }r_{jj^{\prime }}^{\left(\text{obs}\right)}\overset{3}{\underset{d=1}{\sum }}\int_{T_{\alpha }}\gamma_{s}\left(x-z_{j}\right)N_{T_{\alpha }}^{\left(d\right)}\left(x\right)d\omega_{T_{\alpha }}\left(x\right)\\ & \;\times \int_{T_{\alpha }}\gamma_{s}\left(x-z_{j^{\prime }}\right)N_{T_{\alpha }}^{\left(d\right)}\left(x\right)d\omega_{T_{\alpha }}\left(x\right). \end{array}$$

Let us analyze the first term. We have

(21) $$\begin{array}{cc} & \underset{j,j^{\prime }\in J}{\sum }r_{j,j^{\prime }}^{\left(\text{obs}\right)}\overset{3}{\underset{d=1}{\sum }}\int_{S}\gamma_{s}\left(x-z_{j}\right)N_{S}^{\left(d\right)}\left(x\right)d\omega_{S}\left(x\right)\\ & \;\;\;\;\;\times \int_{S}\gamma_{s}\left(x-z_{j^{\prime }}\right)N_{S}^{\left(d\right)}\left(x\right)d\omega_{S}\left(x\right)\\ & \;\;=\underset{j,j^{\prime }\in J}{\sum }r_{j,j^{\prime }}^{\left(\text{obs}\right)}\overset{3}{\underset{d=1}{\sum }}\int_{S\times S}\gamma_{s}\left(x-z_{j}\right)N_{S}^{\left(d\right)}\left(x\right)\gamma_{s}\\ & \;\;\;\;\;\;\;\;\;\;\times \left(y-z_{j^{\prime }}\right)N_{S}^{\left(d\right)}\left(y\right)d\omega_{S}\left(x\right)d\omega_{S}\left(y\right)\\ & \;\;=\int_{S\times S}\;\underset{j,j^{\prime }\in J}{\sum }r_{j,j^{\prime }}^{\left(\text{obs}\right)}\gamma_{s}\left(x-z_{j}\right)\gamma_{s}\left(y-z_{j^{\prime }}\right)\\ & \;\;\;\;\;\;\;\times \left(N_{S}\left(x\right)\cdot N_{S}\left(y\right)\right)d\omega_{S}\left(x\right)d\omega_{S}\left(y\right)\\ & \;\;=\int_{S\times S}K_{\text{obs}}\left(x,y\right)\left(N_{S}\left(x\right)\cdot N_{S}\left(y\right)\right)d\omega_{S}\left(x\right)d\omega_{S}\left(y\right), \end{array}$$

with the notation

(22) $$\begin{array}{c} K_{\text{obs}}\left(x,y\right)=\underset{j,j^{\prime }\in J}{\sum }r_{j,j^{\prime }}^{\left(\text{obs}\right)}\gamma_{s}\left(x-z_{j}\right)\gamma_{\text{s}}\left(y-z_{j^{\prime }}\right). \end{array}$$

Treating the other terms similarly, we can rewrite the error term in the form

(23) $$\begin{array}{cc} {ℰ}_{\text{obs}} & =\frac{1}{\sigma^{2}}\int_{S\times S}K_{\text{obs}}\left(x,y\right)\left(N_{S}\left(x\right)\cdot N_{S}\left(y\right)\right)d\omega_{S}\left(x\right)d\omega_{S}\left(y\right)\\ & \;-\frac{2}{\sigma^{2}}\int_{S\times T_{\alpha }}K_{\text{obs}}\left(x,y\right)\\ & \;\;\;\;\times \left(N_{S}\left(x\right)\cdot N_{T_{\alpha }}\left(y\right)\right)d\omega_{S}\left(x\right)d\omega_{T_{\alpha }}\left(y\right)\\ & \;+\frac{1}{\sigma^{2}}\int_{T_{\alpha }\times T_{\alpha }}K_{\text{obs}}\left(x,y\right)\\ & \;\;\;\;\times \left(N_{T_{\alpha }}\left(x\right)\cdot N_{T_{\alpha }}\left(y\right)\right)d\omega_{T_{\alpha }}\left(x\right)d\omega_{T_{\alpha }}\left(y\right) \end{array}$$

and we will abbreviate this (introducing a notation for the right-hand side) as *ℰ*_obs_ = (1/*σ* ^2^)||*S*−*T*_*α*_||_obs_^2^. Thus, we can write

(24) $$\begin{array}{c} p\left(y\;|\;T_{\alpha },\sigma \right)=\text{cst}\;\exp\;\left(-\frac{1}{2\sigma^{2}}{||S-T_{\alpha }||}_{\text{obs}}^{2}\right). \end{array}$$

We have the following important proposition.

**Proposition 1**

Assume that *γ*_*s*_ is taken equal to the Green function of *L*_*obs*_. Then, when the grid *J* becomes finer and *K*_*obs*_ is given by (22), one has

(25) $$\begin{array}{c} K_{\text{obs}}\left(x,y\right)\to \gamma_{obs}\left(x-y\right). \end{array}$$

See the appendix for a proof of this proposition (which requires some background on the theory of Hilbert spaces with a reproducing kernel) and possible extensions. For practical purposes, we will use *γ*_obs_ instead of *K*_obs_ in ||·||_obs_, therefore assuming that the sensors are chosen according to the proposition. It is interesting to notice that the resulting norm in this case is precisely the norm that has been introduced in [16] to compare surfaces, when they are considered as elements of a reproducing kernel Hilbert space of *currents*. We refer to [16] for details on the mathematical construction.

 In the rest of the paper, we develop a parametric procedure to estimate the template *T* from the observation of i.i.d. surfaces *S* ^(1)^, *S* ^(2)^,…, *S* ^(*N*)^ generated as described above. This includes in particular *N* hidden deformation momenta *α* ^(1)^, *α* ^(2)^,…, *α* ^(*N*)^, such that the complete distribution of observed and unobserved variables is

(26) $$\begin{array}{cc} & p\left(\mu ,\alpha^{\left(1\right)},S^{\left(1\right)},\ldots ,\alpha^{\left(N\right)},S^{\left(N\right)}\right)\\ & =\text{cst}\;\exp\;\left(-\frac{\lambda }{2}{||\mu ||}_{T_{0}}^{2}-\frac{1}{2}\overset{N}{\underset{n=1}{\sum }}\left({||\alpha^{\left(n\right)}||}_{T}^{2}+\frac{1}{\sigma^{2}}{||S^{\left(n\right)}-T_{\alpha^{\left(n\right)}}||}_{\text{obs}}^{2}\right)\right), \end{array}$$

where we have written for short *T* = *T*_0,*μ*_.

We now discuss the estimation of the parameter *μ*, and of the associated template *T*_0,*μ*_, which is the main purpose of this paper. This will be implemented with a mode approximation of the EM algorithm, as described in the next section.

## 4. Algorithms for Surface Template Estimation

### 4.1. Mode Approximation EM

Let Θ = (*α* ^(1)^, *α* ^(2)^,…, *α* ^(*N*)^) be the hidden part of the process, representing the collection of initial momenta, and let **S** = (*S* ^(1)^, *S* ^(2)^,…, *S* ^(*N*)^) be the collection of observed surfaces. The complete distribution for the process, including the prior is given by (26).

The EM algorithm is an iterative method that updates a current estimation of *μ* using the following two *E*- and *M*-steps.

*E*-step: determine the conditional expectation $\tilde{\mu }\mapsto E_{\mu }\{\log\;\pi \left(\tilde{\mu },\Theta ,S\right)|S)\}$.

*M*-step: maximize this expression with respect to $\tilde{\mu }$ and replace the current estimation of *μ* by the obtained maximizer.

The conditional expectation can be expanded as

(27) $$\begin{array}{cc} & E_{\mu }\left\{\log\;\pi \left(\tilde{\mu },\Theta ,S\right)\;|\;S)\right\}\\ & \;=-\frac{\lambda }{2}{||\tilde{\mu }||}_{T_{0}}^{2}-\frac{1}{2}\overset{N}{\underset{n=1}{\sum }}E_{\mu }\left({||\alpha^{\left(n\right)}||}_{T}^{2}+\frac{1}{\sigma^{2}}{||S^{\left(n\right)}-T_{\alpha^{\left(n\right)}}||}_{\text{obs}}^{2}\;|\;S^{\left(n\right)}\right).\; \end{array}$$

Considering the highly nonlinear relation between *α* ^(*n*)^ and the deformed surface *T*_*α*^(*n*)^_, an explicit computation in (27) is impossible. We will therefore rely on the classic mode approximation in the EM which replaces the conditional distribution by a Dirac measure taken at the conditional mode, yielding

(28) $$\begin{array}{cc} & E_{\mu }\left\{\log\;\pi \left(\tilde{\mu },\Theta ,S\right)\;|\;S)\right\}\\ & \;\;\approx \frac{\lambda }{2}{||\tilde{\mu }||}_{T_{0}}^{2}+\frac{1}{2}\overset{N}{\underset{n=1}{\sum }}{||{\hat{\alpha }}^{\left(n\right)}||}_{T}^{2}+\frac{1}{\sigma^{2}}{||S^{\left(n\right)}-T_{{\hat{\alpha }}^{\left(n\right)}}||}_{\text{obs}}^{2}\}, \end{array}$$

where

(29) $$\begin{array}{c} {\hat{\alpha }}^{\left(n\right)}=\underset{\alpha }{\arg\;\min\;}\left\{{||\alpha ||}_{T}^{2}+\frac{1}{\sigma^{2}}{||S^{\left(n\right)}-T_{\alpha }||}_{\text{obs}}^{2}\right\}. \end{array}$$

This results in a maximum a posteriori procedure that maximizes alternatively in *μ* and in the *α* ^(*n*)^'s. Like in [15], we will refer to it as a mode approximation to the EM (MAEM) rather than a MAP algorithm, in order to strengthen the fact that it is an approximation of the maximum a posteriori procedure relying on the likelihood of the observed data only. As illustrated in [14], the MAEM can be biased (leading to inexact estimation of the template, even with a very large number of samples), especially when the noise is important, but it is obviously more feasible than the exact EM. Notice that the MAEM method is a special form of EM algorithm, and as such optimizes a lower bound of the log likelihood of the observed data.

We summarize the two steps of the *i*th iteration of the MAEM in our case. Suppose *μ* and the *α* ^(*n*)^'s are the current variables to be updated. Then the next iteration is as follows. 

MAE step: with *μ* (and therefore *T*) fixed, find, for *n* = 1,…, *N*, ${\hat{\alpha }}^{\left(n\right)}$ to minimize

(30) $$\begin{array}{c} {||\alpha ||}_{T}^{2}+\frac{1}{\sigma^{2}}{||S^{\left(n\right)}-T_{\alpha }||}_{\text{obs}}^{2} \end{array}$$

and replace *α* ^(*n*)^ by ${\hat{\alpha }}^{\left(n\right)}$. 

*M* step: with *α* ^(*n*)^ fixed, update *μ* with the minimizer of

(31) $$\begin{array}{c} \lambda {||\tilde{\mu }||}_{T_{0}}^{2}+\overset{N}{\underset{n=1}{\sum }}\left({||\alpha^{\left(n\right)}||}_{T_{0,\tilde{\mu }}}^{2}+\frac{1}{\sigma^{2}}{||S^{\left(n\right)}-{\left(T_{0\tilde{\mu }}\right)}_{\alpha^{\left(n\right)}}||}_{\text{obs}}^{2}\right). \end{array}$$

We now discuss how each of these steps can be implemented.

### 4.2. MAE Step

Our goal in this section is to optimize (30). We work with fixed *n* and drop it from the notation to simplify the expressions. The objective function is

(32) $$\begin{array}{c} E\left(\alpha \right)={||\alpha ||}_{T}^{2}+\frac{1}{\sigma^{2}}{||S-T_{\alpha }||}_{\text{obs}}^{2}. \end{array}$$

This problem is equivalent to the surface matching algorithm considered in [16], with a slightly different formulation since [16] optimize an energy with respect to a time-dependent momentum instead of just the initial momentum (i.e., they solved simultaneously the geodesic estimation and the matching problems). These two formulations are equivalent when using continuous time (they produce the same minima), but they yield different results when discretized. In our setting, formulating the problem as in (32) is natural, and focuses on the modeled random variable, *α*.

We need to compute the variation of *E* with respect to *α*. This computation will be useful for the *M*-step also. We first discuss the discretization of the error term, which follows [16]. Let *S* be a triangulated surfaces, with vertices *x* ^(*S*)^ = (*x*_1_^(*S*)^,…, *x*_*L*_^(*S*)^) and faces *F* ^(*S*)^ = (*f*_1_^(*S*)^,…, *f*_*M*_^(*S*)^). Each face is represented by an ordered triple of vertices: *f* = (*x*_1_^*f*^, *x*_2_^*f*^, *x*_3_^*f*^), and we define the face centers and area-weighted normals by

(33) $$\begin{array}{c} c_{f}=\frac{1}{3}\left(x_{1}^{f}+x_{2}^{f}+x_{3}^{f}\right),\\ N_{f}=\frac{1}{2}\left(x_{2}^{f}-x_{1}^{f}\right)\times \left(x_{3}^{f}-x_{1}^{f}\right). \end{array}$$

Then a discrete approximation of ||*S*−*S*′||_obs_^2^ is

(34) $$\begin{array}{cc} U\left(x^{S^{\prime }}\right) & =\underset{f,f^{\prime }\in F^{\left(S\right)}}{\sum }\gamma_{\text{obs}}\left(c_{f}-c_{f^{\prime }}\right)\left(N_{f}\cdot N_{f^{\prime }}\right)\\ & \;+\underset{f,f^{\prime }\in F^{\left(S^{\prime }\right)}}{\sum }\gamma_{\text{obs}}\left(c_{f}-c_{f^{\prime }}\right)\left(N_{f}\cdot N_{f^{\prime }}\right)\\ & \;-2\underset{f\in F^{\left(S\right)},f^{\prime }\in F^{\left(S^{\prime }\right)}}{\sum }\gamma_{\text{obs}}\left(c_{f}-c_{f^{\prime }}\right)\left(N_{f}\cdot N_{f^{\prime }}\right), \end{array}$$

where *S* is considered as fixed and *U* is therefore considered as a function of the vertices, *x* ^(*S*′)^, of *S*′.

With this notation, we can write

(35) $$\begin{array}{c} E\left(\alpha \right)={||\alpha ||}_{T}^{2}+\frac{1}{\sigma^{2}}U\left(x^{\left(T_{\alpha }\right)}\right). \end{array}$$

We want to compute the gradient of *E* and for this, apply the chain rule to the transformations *α* → *T*_*α*_ → *U*(*x* ^(*T*_*α*_)^), yielding

(36) $$\begin{array}{c} \partial_{\alpha }U={\left(\frac{dx^{\left(T_{\alpha }\right)}}{d\alpha }\right)}^{*}\partial_{x}U, \end{array}$$

where A* is the transpose matrix of *A*.

The gradient of *U* with respect to *x* ^(*S*′)^ has been computed in [16], and is given as follows. We denote by *F*_*l*_^(*S*)^ the set of faces (triangles) that contain a vertex *x*_*l*_ in a triangulated surface *S*. For *f* ∈ *F*_*l*_^(*S*)^, we let *e*_*l*_(*f*) denote the edge opposed to *x*_*l*_ in *f*, positively oriented in *f*. With *S*′ = *T*_*α*_, we have

(37) $$\begin{array}{cc} & \frac{\partial U}{\partial x_{l}^{\left(S^{\prime }\right)}}\left(x^{\left(S^{\prime }\right)}\right)\\ & \;=\underset{f\in F_{l}^{\left(S^{\prime }\right)}}{\sum }\;\underset{g\in F^{\left(S\right)}\cup F^{\left(S^{\prime }\right)}}{\sum }a_{g}(\frac{2}{3}\nabla \gamma_{\text{obs}}\left(c_{f}-c_{g}\right)\left(N_{f}\cdot N_{g}\right)\\ & \;\;\;\;\;\;\;\;\;\;+\gamma_{\text{obs}}\left(c_{f}-c_{g}\right)e_{l}\left(f\right)\times N_{g}), \end{array}$$

where *a*_*g*_ = 1 if *g* ∈ *F* ^(*S*′)^ and *a*(*g*) = −1 if *g* ∈ *F* ^(*S*)^.

Now let us derive the variation of *x* ^(*T*_*α*_)^ with respect to the initial momentum, *α*. We know that *x* ^(*T*_*α*_)^ = *x*(1), where *x* and *a* evolve according to the system (7)

(38) $$\begin{array}{c} \frac{\text{d}x_{k}}{\text{d}t}=\overset{L}{\underset{l=1}{\sum }}\gamma_{kl}a_{l},\;\;\frac{\text{d}a_{k}}{\text{d}t}=-2\overset{L}{\underset{l=1}{\sum }}\gamma_{kl}^{\prime }\left(a_{l}\cdot a_{k}\right)\left(x_{k}-x_{l}\right) \end{array}$$

with *x*(0) = *x* ^(*T*)^ and *a*(0) = *α* (and *γ*_*kl*_, *γ*_*kl*_′ are short for *γ*(||*x*_*k*_−*x*_*l*_||^2^) and *γ*′(||*x*_*k*_−*x*_*l*_||^2^)). Now an infinitesimal variation *α* → *α* + *δ* *α* in the initial condition induces infinitesimal variations *a* + *δ* *a* and *x* + *δ* *x* over time, and the pair (*δ* *x*, *δ* *a*) obeys the following differential system, that can be obtained from a formal differentiation of (7):

(39) $$\begin{array}{cc} \frac{\text{d}\delta x_{k}\left(t\right)}{\text{d}t} & =\overset{L}{\underset{l=1}{\sum }}\gamma_{kl}\delta a_{l}+2\overset{L}{\underset{l=1}{\sum }}\gamma_{kl}^{\prime }a_{l}\left(x_{k}-x_{l}\right)\cdot \left(\delta x_{k}-\delta x_{l}\right), \end{array}$$

(40) $$\begin{array}{cc} \frac{\text{d}\delta a_{k}\left(t\right)}{\text{d}t} & =-2\overset{L}{\underset{l=1}{\sum }}\gamma_{kl}^{\prime }\left(a_{l}\cdot \delta a_{k}+\delta a_{l}\cdot a_{k}\right)\left(x_{k}-x_{l}\right)\\ & \;-2\overset{L}{\underset{l=1}{\sum }}\gamma_{kl}^{\prime }\left(a_{l}\cdot a_{k}\right)\left(\delta x_{k}-\delta x_{l}\right)\\ & \;-4\overset{L}{\underset{l=1}{\sum }}\gamma_{kl}^{\prime \prime }\left(a_{l}\cdot a_{k}\right)\left(x_{k}-x_{l}\right)\left(\left(x_{k}-x_{l}\right)\cdot \left(\delta x_{k}-\delta x_{l}\right)\right) \end{array}$$

with *γ*_*kl*_′′ = *γ*′′(||*x*_*k*_−*x*_*l*_||^2^).

One can rewrite it in the matrix form:

(41) $$\begin{array}{c} \frac{\text{d}}{\text{d}t}\left(\begin{array}{c} \delta x\\ \delta \alpha \end{array}\right)=J\left(t\right)\left(\begin{array}{c} \delta x\\ \delta \alpha \end{array}\right), \end{array}$$

where $J\left(t\right)=\left(\begin{array}{cc} J_{xx} & J_{xa}\\ J_{ax} & J_{aa} \end{array}\right)$ with

(42) $$\begin{array}{cc} J_{xx}\left(k,l\right) & =\left(2\overset{L}{\underset{q=1}{\sum }}\gamma_{kq}^{\prime }a_{q}{\left(x_{l}-x_{q}\right)}^{*}\right)\delta_{kl}-2\gamma_{kl}^{\prime }a_{l}{\left(x_{k}-x_{l}\right)}^{*}\\ J_{xa}\left(k,l\right) & =\gamma_{kl}Id_{{\mathbb{R}}^{3}}.\\ J_{ax}\left(k,l\right) & =-(2\overset{L}{\underset{q=1}{\sum }}\left(a_{q}\cdot a_{l}\right)\\ & \;\;\times \left(\gamma_{kq}^{\prime }Id_{{\mathbb{R}}^{3}}+2\gamma_{kq}^{\prime \prime }\left(x_{l}-x_{q}\right){\left(x_{l}-x_{q}\right)}^{*}\right)\delta_{kl})\\ & \;+2\left(a_{l}\cdot a_{k}\right)\left(\gamma_{kl}^{\prime }Id_{{\mathbb{R}}^{3}}+2\gamma_{kl}^{\prime \prime }\left(x_{k}-x_{l}\right){\left(x_{k}-x_{l}\right)}^{*}\right).\\ J_{aa}\left(k,l\right) & =-\left(2\left(\overset{L}{\underset{q=1}{\sum }}\gamma_{kq}^{\prime }\left(x_{l}-x_{q}\right)a_{q}^{*}\right)\right)\delta_{kl}-2\gamma_{kl}^{\prime }\left(x_{k}-x_{l}\right)a_{k}^{*}. \end{array}$$

Solving this system with initial condition *δ* *x*(0) = 0 and *δ* *a*(0) = *δ* *α* provides what we have denoted

(43) $$\begin{array}{c} \left(\frac{dx^{\left(T_{\alpha }\right)}}{\text{d}\alpha }\right)\delta \alpha . \end{array}$$

One does not need to compute all the coefficients of the matrix *d* *x* ^(*T*_*α*_)^/*d* *α* using this equation in order to apply the transpose in (36). This is fortunate because this would constitute a computationally demanding effort given that this matrix is 3*L* by 3*L* with *L* large. The right hand side of (36) can be in fact computed directly using a single dynamical system, given by

(44) $$\begin{array}{c} \frac{\text{d}}{\text{d}t}\left(\begin{array}{c} \eta_{x}\\ \eta_{\alpha } \end{array}\right)=-J{\left(t\right)}^{*}\left(\begin{array}{c} \eta_{x}\\ \eta_{\alpha } \end{array}\right), \end{array}$$

where *J*(*t*) is defined in (39). If (44) is solved from time *t* = 1 to time *t* = 0 with *η*_*x*_(1) = ∂_*x*_ *U* and *η*_*α*_(1) = 0, then

(45) $$\begin{array}{c} \partial_{\alpha }U=\eta_{\alpha }\left(0\right). \end{array}$$

This is a simple consequence of the theory of linear differential systems (a proof is provided in the Appendix for completeness). Note that the matrix *J*(*t*) depends on the solution of (7) computed with initial conditions *x*(0) = *x* ^(*T*)^ and *a*(0) = *α*. To emphasize this dependency, we will denote it *J*(*t*) = *J* ^(*T*,*α*)^(*t*) in the following.

Given this, we see that a variation *α* → *α* + *δ* *α* induces a first-order variation *δ* *E* of the energy given by

(46) $$\begin{array}{c} \delta E=2{\langle \delta \alpha \;,\;\alpha \rangle }_{T}+\frac{1}{\sigma^{2}}\delta \alpha \cdot \eta_{\alpha }\left(0\right), \end{array}$$

where the *T*-dot product is 〈*δ* *α* , *α*〉_*T*_ = *δ* *α* · (Γ_*T*_ *α*) and Γ_*T*_ is the matrix formed with 3 by 3 blocs *γ*_*kl*_ *I* *d*_ℝ^3^_.

We choose to operate the gradient descent with respect to this dot product and therefore choose a variation proportional to *δ* *α* = −(2*α* + Γ_*T*_^−1^ *η*_*α*_(0)). So, the algorithm to compute an optimal *α* is the following.

**Algorithm 1 (MAE Step for Surface Template Estimation)**

1. Compute the variation ∂_*x*^(*T*_*α*_)^_ *U* using (37).
2. Solve backward in time (44) initialized with *η*_*x*_(1) = ∂_*x*^(*T*_*α*_)^_ *U* and *η*_*α*_ = 0.
3. Replace *α* by *α* − *ϵ*(2*α* + (1/*σ* ^2^)Γ_*T*_^−1^ *η*_*α*_(0)) (using a line-search to optimize *ϵ*).

This algorithm has to be applied *N* times (for all *α*_*k*_, *k* = 1,…, *N*) in the MAE step.

**Remark 1**

The matrix Γ_*T*_ being typically very badly conditioned, we numerically compute Γ_*T*_^−1^ *η*_*α*_ after adding a small positive number to the diagonal of Γ_*T*_. The inversion itself is computed using conjugate gradient.

### 4.3. M Step

There are many similarities between the *M*-step and the *E*-step variational problems, so that we will be able to only sketch the detail of the computation here. We need to minimize

(47) $$\begin{array}{c} \tilde{E}\left(\mu \right)={||\mu ||}_{T_{0}}^{2}+\frac{1}{\lambda }\overset{N}{\underset{n=1}{\sum }}{\tilde{U}}^{\left(n\right)}\left(x^{\left(T_{0,\mu }\right)}\right) \end{array}$$

with

(48) $$\begin{array}{c} {\tilde{U}}^{\left(n\right)}\left(x^{\left(T\right)}\right)={||\alpha^{\left(n\right)}||}_{T}^{2}+\frac{1}{\sigma^{2}}{||S-T_{\alpha^{\left(n\right)}}||}_{\text{obs}}^{2}. \end{array}$$

Let us consider the variation of each term in the sum (fixing *n*, that we temporarily drop from the notation). Since

(49) $$\begin{array}{c} {||\alpha ||}_{T}^{2}=\overset{L}{\underset{k,l=1}{\sum }}\gamma \left({||x_{k}^{\left(T\right)}-x_{l}^{\left(T\right)}||}^{2}\right)\left(\alpha_{k}\cdot \alpha_{l}\right), \end{array}$$

we can write

(50) $$\begin{array}{c} \frac{\partial {||\alpha ||}_{T}^{2}}{\partial x_{k}^{\left(T\right)}}=2\overset{L}{\underset{l=1}{\sum }}\gamma^{\prime }\left({||x_{k}^{\left(T\right)}-x_{l}^{\left(T\right)}||}^{2}\right)\left(\alpha_{k}\cdot \alpha_{l}\right)\left(x_{k}^{\left(T\right)}-x_{l}^{\left(T\right)}\right). \end{array}$$

The function *U* being defined as before, we see that the derivative of the second term is given by applying the chain rule again, this time in the form

(51) $$\begin{array}{c} {\left(\frac{dx^{\left(T_{\alpha }\right)}}{dx^{\left(T\right)}}\right)}^{*}\partial_{x^{\left(T_{\alpha }\right)}}U. \end{array}$$

Like in the previous section, the transpose of the differential applied to the gradient of *U* can be computed by solving a dynamical system backward in time. In fact, it is the same system as with the variation in *α*, namely,

(52) $$\begin{array}{c} \frac{\text{d}}{\text{d}t}\left(\begin{array}{c} \eta_{x}\\ \eta_{\alpha } \end{array}\right)=-J^{\left(T,\alpha \right)}{\left(t\right)}^{*}\left(\begin{array}{c} \eta_{x}\\ \eta_{\alpha } \end{array}\right) \end{array}$$

still initialized with *η*_*x*_(1) = ∂_*x*^(*T*_*α*_)^_ *U* and *η*_*α*_(1) = 0, but the relevant result now is *η*_*x*_(0). The gradient of $\tilde{U}$ is then

(53) $$\begin{array}{c} \partial_{x^{\left(T\right)}}U=\partial_{x^{\left(T\right)}}{||\alpha ||}_{T}^{2}+\frac{1}{\sigma^{2}}\eta_{x}\left(0\right). \end{array}$$

Once this is computed, the next step is to compute (reintroducing *n* in the notation)

(54) $$\begin{array}{c} {\left(\frac{dx^{\left(T_{0\mu }\right)}}{d\mu }\right)}^{*}\left(\overset{N}{\underset{n=1}{\sum }}\partial_{x^{\left(T\right)}}U^{\left(n\right)}\right). \end{array}$$

This follows a similar procedure, using (44), with *T*_0_ instead of *T* and *μ* instead of *α*. This requires solving

(55) $$\begin{array}{c} \frac{\text{d}}{\text{d}t}\left(\begin{array}{c} \eta_{x}\\ \eta_{\mu } \end{array}\right)=-J^{\left(T_{0},\mu \right)}{\left(t\right)}^{*}\left(\begin{array}{c} \eta_{x}\\ \eta_{\mu } \end{array}\right), \end{array}$$

initialized with $\eta_{x}(1)=\partial_{x^{\left(T\right)}}\tilde{U}$ and *η*_*μ*_(1) = 0. The variation of $\tilde{E}$ associated to an infinitesimal variation of *μ* is then

(56) $$\begin{array}{cc} \delta E & ={\langle \delta \mu ,\;2\mu \rangle }_{T_{0}}+\frac{1}{\lambda }\left(\delta \mu \cdot \eta_{\mu }\left(0\right)\right)\\ & =\langle \delta \mu ,\;2\mu +\frac{1}{\lambda }\Gamma_{T_{0}}^{-1}\eta_{\mu }\left(0\right)\rangle . \end{array}$$

We summarize the M step in the following algorithm.

**Algorithm 2 (*M*-Step Algorithm for Surface Template Estimation)**

(1) For *n* = 1,…, *N*:
  (1.1) Compute ∂_*x*^(*T*_*α*^(*n*)^_)^_ *U* using (37).
  (1.2) Solve system (44) backward in time with initial condition *η*_*x*_^(*n*)^(1) = ∂_*x*^(*T*_*α*^(*n*)^_)^_ *U* and *η*_*α*_^(*n*)^(1) = 0.
  (1.3) Compute
    (57) $$\begin{array}{c} \partial_{x^{\left(T\right)}}{\tilde{U}}^{\left(n\right)}=\partial_{x^{\left(T\right)}}{||\alpha^{\left(n\right)}||}_{T}^{2}+\frac{1}{\sigma^{2}}\eta_{x}^{\left(n\right)}\left(0\right) \end{array}$$
    using (50).
(2) Solve system (55) backward in time with
  (58) $$\begin{array}{c} \eta_{x}\left(1\right)=\overset{N}{\underset{n=1}{\sum }}\partial_{x^{\left(T\right)}}{\tilde{U}}^{\left(n\right)} \end{array}$$
  and *η*_*μ*_(1) = 0.
(3) Replace *μ* by *μ* − *ϵ*(*μ* + Γ_*T*_0__^−1^ *η*_*μ*_(0)/*λ*), *ϵ* being optimized with a line search.

### 4.4. Surface Template Estimation Algorithm

We finally summarize the surface template estimation algorithm:

**Algorithm 3 (Surface Template Estimation)**

Having the hypertemplate *T*_0_ and observed surfaces *S* ^(1)^,…, *S* ^(*N*)^, the goal is to estimate the template *T*. Let *T*, *μ*, *α* ^(1)^,…, *α* ^(*N*)^ denote the current estimation with initial guess *T* = *T*_0_, *μ* = 0, *α* ^(*n*)^ = 0. Then, in the next iteration, update with the following steps:

- With *T* fixed, apply Algorithm 1 to update each *α* ^(*n*)^, *n* = 1,…, *N*.
- With *α* ^(*n*)^'s fixed, apply Algorithm 2 to obtain a new value for *μ*.
- Solve (7) initialized with the hypertemplate *T*_0_ and the newly obtained *μ* to update the estimated template, *T*.

## 5. Result and Discussion

We applied the algorithm to surface data of human brain's caudate, thalamus, and hippocampus. All data are courtesy of Center for Imaging Science at Johns Hopkins University. Each surface has around 5–10 thousand triangle cells. We randomly chose one as the hypertemplate and the others as observed surfaces. In these experiments, we set *λ* = 1.0 and *σ* ^2^ = 1.0.

**Figure 2:**
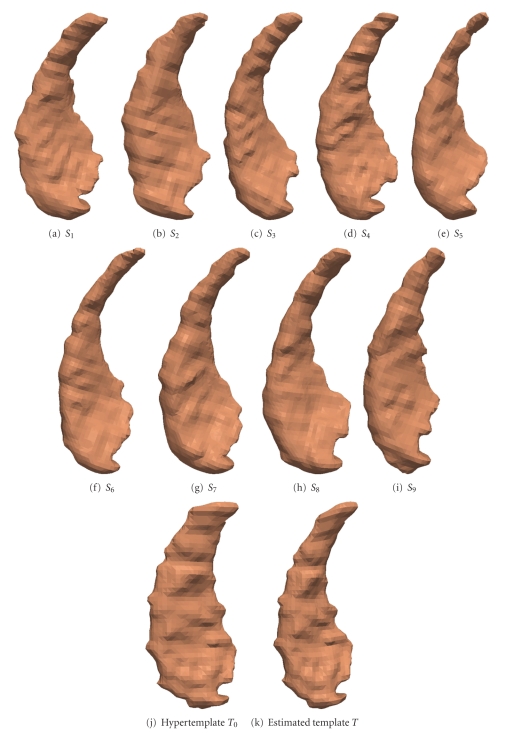
Estimating the surface template from 9 caudate data. (a)–(i) observed surfaces. (j) is the hypertemplate. (k) is the result.

**Figure 3:**
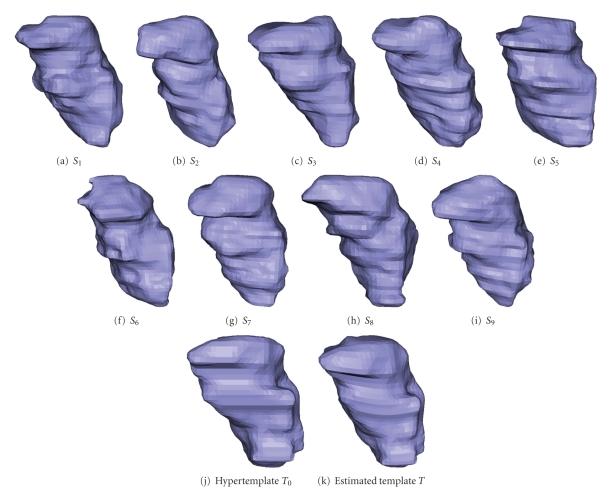
Estimating the surface template from 9 thalamus data. (a)–(i) observed surfaces. (j) is the hypertemplate. (k) is the result.

Figures 2 and 3 are the template estimation result for caudate and thalamus, respectively.

**Figure 4:**
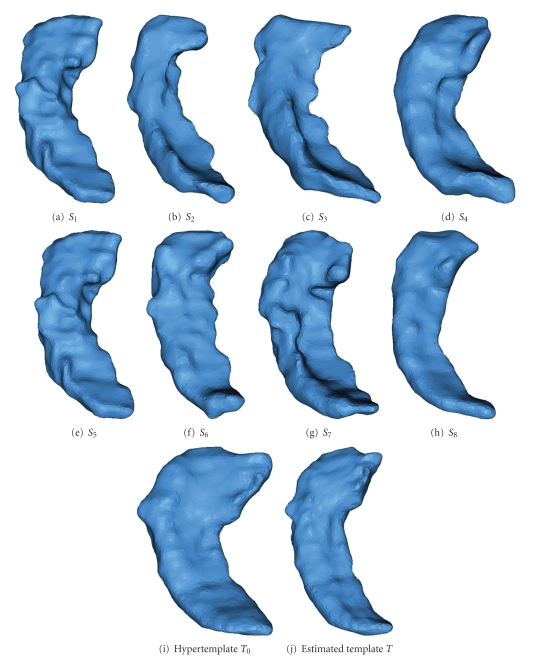
Estimating the surface template from 101 data. (a)–(h) are 8 examples out of 101 observed surfaces. (i) is the hypertemplate. (j) is the result.

We also applied the algorithm to 101 hippocampus surface data in the BIRN Project (Biomedical Informatics Research Network). In Figure 4, Panels (a)–(h) are 8 examples of the 101 observations. Panel (i) is the hypertemplate and Panel (j) is the estimated template. 

The result is visually satisfying in the sense that the estimated template is found to agree with a qualitative representation of the population. For example, in the caudate experiment, the estimated template has an obviously narrower upper tip than the hypertemplate. This captures the population characteristic since most observed data have narrower upper tips. Notice that the obtained template does not look smoother than the rest of the population, as would typically yield template estimation methods that average over fixed parametrizations.

**Figure 5:**
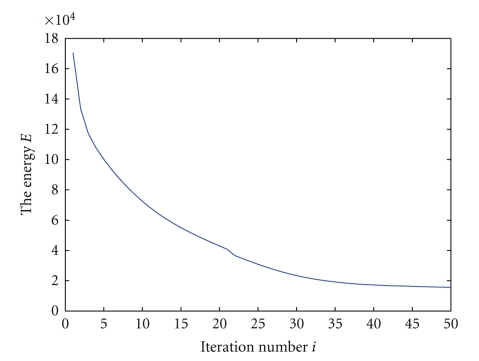
Energy change with the iteration.

Figure 5 shows how the energy in (31) changes with the iteration in the caudate experiment. This confirms the effectiveness and convergence of the algorithm. One can see the energy drops quickly in the first twenty iterations, then gradually slows down. After the 35th iteration, the energy changes little and the estimated template remains stable.

**Figure 6:**
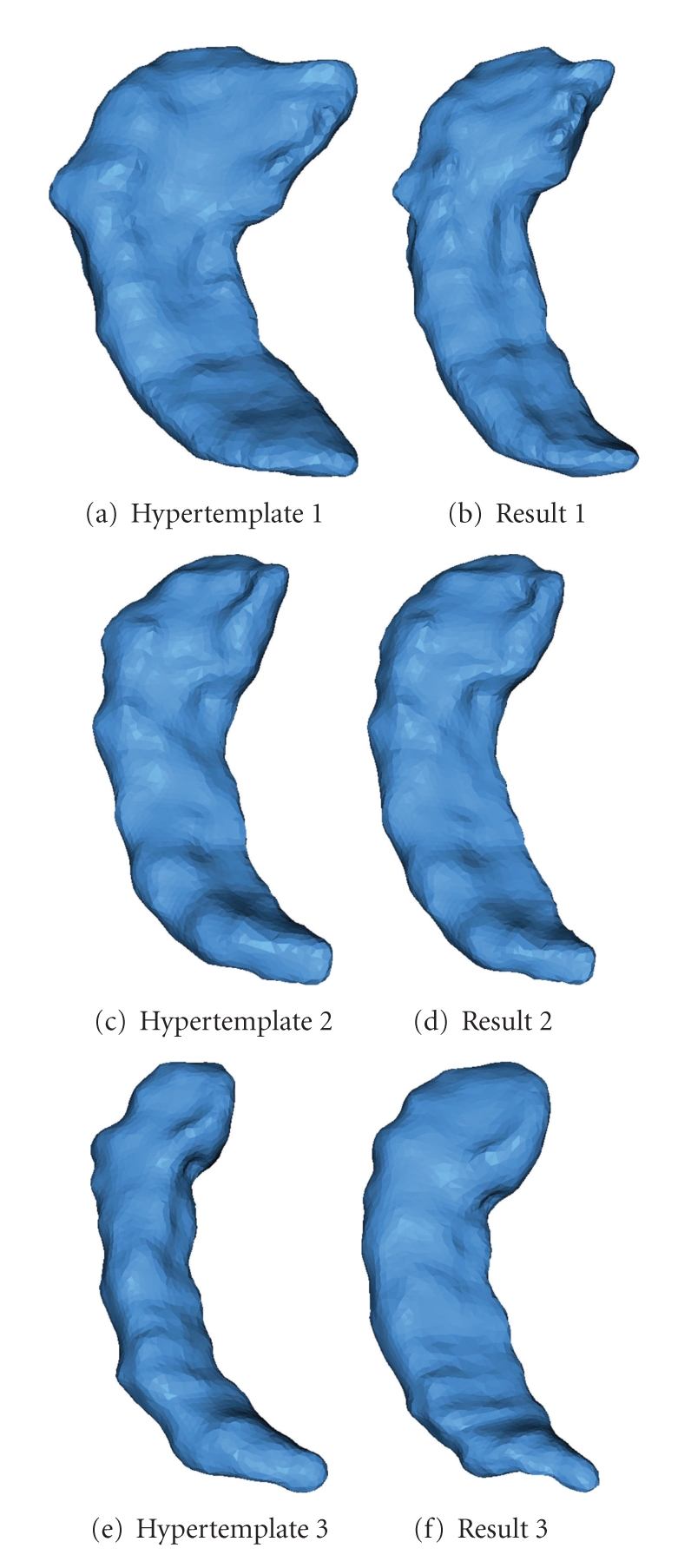
For the same observed population, we choose different surfaces as hypertemplate. The results only have minor differences.

In our model, the hypertemplate can be provided by an atlas obtained from other studies, although we here simply choose one of the surfaces in the population. Actually, as Figure 6 shows, different choices for the hypertemplate yield very similar results.

## 6. Conclusion

In this paper, we have presented a Bayesian approach for surface template estimation. We have built, for this purpose, a generative statistical model for surfaces: the construction first applies a random initial momentum to the template surface, then assumes an observation process using test functions and noise. The template is assumed to be generated as a random deformation of a *hypertemplate*, completing the Bayesian model. We used a mode approximation EM scheme to estimate the surface template, and introduced for this purpose a novel surface matching algorithm optimizing with respect to the initial momentum. The procedure has been tested with caudate, thalamus, and hippocampus surface data, showing its effectiveness and convergence, and also experimentally proved to be robust to variations in the choice of the hypertemplate.

## Appendices

### A. Proof of Proposition 1

Let us assume that *L*_obs_ *γ*_*s*_ = *δ*_0_, that is, *γ*_*s*_ is the Green Kernel of the operator *L*_obs_. This assumption implies, in particular, that *γ*_*s*_ = *γ*_obs_. Given a smooth function *f*, denote

(A.1) $$\begin{array}{c} f_{J}\left(x\right)=\underset{j,j^{\prime }\in J}{\sum }r_{j,j^{\prime }}^{\left(\text{obs}\right)}\gamma_{\text{obs}}\left(x-z_{j}\right)f\left(z_{j^{\prime }}\right). \end{array}$$

Define the vector space

(A.2) $$\begin{array}{c} V_{J}=\left\{g\in V:g\left(x\right)=\underset{j\in J}{\sum }\gamma_{\text{obs}}\left(x-z_{j}\right)\rho_{j},\;\rho_{j}\in \mathbb{R},\;j\in J\right\}. \end{array}$$

Introduce the RKHS *V* with scalar product given by

(A.3) $$\begin{array}{c} {\langle w\;,\;w^{\prime }\rangle }_{V}=\int_{{\mathbb{R}}^{d}}\left(L_{\text{obs}}w\right)w^{\prime }dx. \end{array}$$

Then *f*_*J*_ can be identified to the orthogonal projection of *f* on *V*_*j*_, because *r* ^(obs)^ is the inverse of the Gram matrix (matrix of dot products) of (*γ*_obs_(·−*z*_*j*_), *j* ∈ *J*), which are the generators of *V*_*J*_, and

(A.4) $$\begin{array}{c} f\left(z_{j}\right)={\langle f\;,\;\gamma_{\text{obs}}\left(\cdot ,z_{j}\right)\rangle }_{V} \end{array}$$

by assumption.

Now, let *J*_*m*_ be a sequence of progressively finer grids with resolution *ϵ*_*m*_ tending to 0 when *m* tends to infinity. We prove that ||*f*_*J*_*m*__−*f*||_*V*_ → 0, for which it suffices to prove that ⋃*V*_*J*_*m*__ is dense in *V*. This is equivalent to the fact that no nonzero *g* in *V* can be orthogonal to all the *V*_*J*_*m*__'s. But *g* orthogonal to *V*_*J*_*m*__ is only possible when, for all *j* ∈ *J*_*m*_,

(A.5) $$\begin{array}{c} g\left(z_{j}\right)={\langle g,\;\gamma_{w}\left(\cdot -z_{j}\right)\rangle }_{V}=0. \end{array}$$

Since the *z*_*j*_′*s* form an arbitrarily fine grid in *V* and functions in *V* are continuous, this implies *g* = 0.

So, *f*_*J*_ → *f* in *V*, which implies, for example, pointwise convergence as soon as *V* is embedded in the set of continuous functions (that is, if *γ*_obs_ is continuous). This directly implies Proposition 1 in the case *γ*_*s*_ = *γ*_obs_, by taking *f*(*x*) = *γ*_obs_(*x* − *y*) for a given *y*.

Extensions of this result is when *γ*_*s*_ is obtained by applying some linear operator, say *A*, to *γ*_obs_. One then has

(A.6) $$\begin{array}{c} K_{\text{obs}}\left(\cdot ,\tilde{x}\right)=A\left(\underset{j,j^{\prime }\in J}{\sum }r_{j,j^{\prime }}^{\left(w\right)}\gamma_{\text{obs}}\left(\cdot -z_{j}\right)\gamma_{s}\left(\tilde{x}-z_{j^{\prime }}\right)\right) \end{array}$$

and passing to the limit in the sum (for which one needs *γ*_*s*_ ∈ *V* and *A* continuous on *V*), one gets $K_{\text{obs}}(x,\tilde{x})\to A^{2}\gamma_{\text{obs}}$.

### B. Transposing Linear Differential Equations

We here justify the procedure described in Section 4, and prove the following fact: consider the solution, *z*(*t*) ∈ ℝ^*p*^, of the ordinary differential equation

(B.1) $$\begin{array}{c} \partial_{t}z=J\left(t\right)z, \end{array}$$

where *J* is a time-dependent operator (a *p* by *p* matrix). Then, for any *u* ∈ ℝ^*p*^, we have

(B.2) $$\begin{array}{c} u\cdot z\left(1\right)=\eta \left(0\right)\cdot z\left(0\right), \end{array}$$

where *η* is the solution of the differential equation

(B.3) $$\begin{array}{c} \partial_{s}\eta =-J{\left(s\right)}^{*}\eta \end{array}$$

with *η*(1) = *u*.

Let us prove this result. First remark that since *z* is the solution of a linear equation, it depends linearly on the initial condition, *z*(0). More precisely, let *M*(*s*, *t*) be the *p* by *p* matrix such that

(B.4) $$\begin{array}{c} \partial_{t}M\left(s,t\right)=J\left(t\right)M\left(s,t\right) \end{array}$$

and *M*(*s*, *s*) = *I* *d*_ℝ^*p*^_. Then *z*(*t*) = *M*(0, *t*)*z*(0), and, obviously, *η*(0) = *M*(0,1)**u*. Using the identity *M*(*t*, *s*)*M*(*s*, *t*) = *I* *d*_ℝ^*p*^_, we obtain

(B.5) $$\begin{array}{cc} 0 & =M\left(t,s\right)\left(\partial_{s}M\left(s,t\right)\right)+\left(\partial_{s}M\left(t,s\right)\right)M\left(s,t\right)\\ & =\left(\partial_{s}M\left(s,t\right)\right)M\left(t,s\right)+J\left(s\right) \end{array}$$

so that ∂_*s*_ *M*(*s*, *t*) = −*M*(*s*, *t*)*J*(*s*). Computing the transposed equation yields and taking *t* = 1

(B.6) $$\begin{array}{c} \partial_{s}M{\left(s,1\right)}^{*}=-J{\left(s\right)}^{*}M{\left(s,1\right)}^{*}. \end{array}$$

Thus, if *η*(*s*) = *M*(*s*,1)**u*, we have *η*(1) = *u* and ∂_*s*_ *η* = −*J*(*s*)**η* as announced.
